# Impact of Visual Context on Public Perceptions of Non-Human Primate Performers

**DOI:** 10.1371/journal.pone.0118487

**Published:** 2015-02-25

**Authors:** Katherine A. Leighty, Annie J. Valuska, Alison P. Grand, Tamara L. Bettinger, Jill D. Mellen, Stephen R. Ross, Paul Boyle, Jacqueline J. Ogden

**Affiliations:** 1 Disney’s Animals, Science and Environment, Walt Disney World, Lake Buena Vista, FL 32830, United States of America; 2 Lemur Conservation Foundation, Myakka City, FL 34251, United States of America; 3 Lester E. Fisher Center for the Study and Conservation of Apes, Lincoln Park Zoo, Chicago, IL 60614, United States of America; 4 Association of Zoos and Aquariums, Silver Spring, MD 20910, United States of America; CNR, ITALY

## Abstract

Prior research has shown that the use of apes, specifically chimpanzees, as performers in the media negatively impacts public attitudes of their conservation status and desirability as a pet, yet it is unclear whether these findings generalize to other non-human primates (specifically non-ape species). We evaluated the impact of viewing an image of a monkey or prosimian in an anthropomorphic or naturalistic setting, either in contact with or in the absence of a human. Viewing the primate in an anthropomorphic setting while in contact with a person significantly increased their desirability as a pet, which also correlated with increased likelihood of believing the animal was not endangered. The majority of viewers felt that the primates in all tested images were “nervous.” When shown in contact with a human, viewers felt they were “sad” and “scared”, while also being less “funny.” Our findings highlight the potential broader implications of the use of non-human primate performers by the entertainment industry.

## Introduction

Non-human primates frequently appear in television, film, and commercial advertising. Their use as performers is most often for comedic purposes [1] and while market research on the placement of animals in advertising is limited, existing studies have found the practice can significantly increase product likeability [2, 3]. As such, there is continued demand for the use of non-human primate performers by these industries. But, over the past decade, scientists involved in the study and conservation of non-human primates have adopted policy statements against these practices, citing that primate performers are frequently denied normal social and psychological development by being taken away from their mothers shortly after birth, may be trained with aversive techniques, are at increased risk of contracting and spreading disease due to their close proximity to humans, and that their biology and conservation status may be inaccurately perceived by the public [4, 5]. This sentiment has also been echoed by professionals in the zoo and aquarium industry with the release of a white paper outlining their stance against the use of apes in the media and for commercial purposes [6].

Recent scientific studies support the philosophy behind these policy/position statements. In a 2008 survey, Ross and colleagues found that people were less likely to perceive chimpanzees as endangered compared to other great apes (gorillas and orangutans). When probed regarding the reasoning behind this belief, responses centered on the fact that chimpanzees are often seen on television, in movies, and appear in print media such as greeting cards and advertisements [7]. Follow-up research conducted using composite images of chimpanzees depicted in a variety of contexts found that showing these animals in the presence of humans in an anthropomorphic environment, such as an office space, had significant impacts on viewers’ attitudes in that they were more likely to believe that wild populations of chimpanzees are stable in number and more likely to consider owning a chimpanzee as a pet [8].

Given the animal welfare concerns around the use of non-human primate performers and the potential for their use to negatively influence public attitudes, The Walt Disney Company, in 2012, led the industry by adding specific language to their company policy on the use of live animals in entertainment which prohibited the use of all apes (chimpanzees, gorillas, orangutans, bonobos, gibbons and siamangs) and other large primates (baboons and macaques) outside of a zoo/sanctuary habitat or natural environment in all of their entertainment ventures [9]. This landmark decision by The Walt Disney Company along with recent pledges not to use great ape performers in their advertising made by other large companies including Dodge Motor Company, Great Clips, Honda, Samsung, Sprint, and Yahoo as well as international advertising agencies including Campbell Ewald, Leo Burnett, Saatchi and Saatchi, and McCann Erickson, have sent a message to the providers of apes for entertainment [10].

While it is clear that attitudes about the use of certain non-human primates (i.e. apes and larger monkeys) as performers are changing, questions remain regarding the impacts of engaging in this practice with other smaller primates such as many of the New World monkeys and prosimians, several of which are relatively common in the North American pet trade and frequently appear in movies, television, commercials, and print media. For example, one capuchin monkey performer, Crystal, has appeared in numerous television sitcoms in the past few years, including multiple episodes of “Family Practice” and “Community”, the Teen Choice Awards, and several feature films including “We Bought a Zoo,” “Zookeeper,” and “The Hangover Part II” [11]. With the aim of determining if and how the use of these other types of non-human primates (specifically capuchin monkeys, squirrel monkeys, and ring-tailed lemurs) as performers influences public attitudes, we surveyed over 1100 people upon viewing a composite image containing one of these primate species, in either an anthropomorphic or naturalistic forest setting, with a human present or absent. Following from the findings of prior research on chimpanzee performers [7,8], we hypothesized that these factors would interact such that primates viewed in a human setting, in the presence of a human, would be perceived to be more stable in their wild populations, more desirable as a pet, and experiencing a negative emotional state.

## Materials and Methods

### Composite Image Construction

Test images were constructed by combining several digital images into a single composite image using image-editing software. Images varied by species (capuchin, lemur, or squirrel monkey), human presence (present and touching the primate or absent), and context (stereotypical office or natural forest setting), resulting in a total of 12 unique images. Capuchin monkeys (*Cebus sp*.) are a medium-sized New World monkey commonly used as performers in the media, squirrel monkeys (*Saimiri sp*.) are a small New World monkey less commonly seen in the media, and ring-tailed lemurs (*Lemur catta*) are a prosimian infrequently in the media. In all images the depicted primates were of roughly the same size, with a neutral expression, and were oriented toward the viewer.

### Survey Administration

The survey was administered to 1144 adult visitors aged 18 and older (708 women, 436 men) at the Lincoln Park Zoo (Chicago, IL) in July and August of 2011. Participants were randomly selected by approaching every fifth person moving through the main mall of the zoo campus. Of all individuals approached, 32.4% declined to participate in which case the data collector thanked them for their time and moved on to approach the subsequent fifth person moving through the area.

Those visitors that agreed to participate were shown one of the composite images, and asked to verbally answer two questions, to which they could respond yes, no or maybe. Aligning with previous research on chimpanzee performers [7,8] the questions were, “Would you consider getting this animal as a pet?” and “Is this species endangered in the wild?” In addition, respondents were asked to circle the word or words that best described the animal in the picture from the following list: “happy, sad, scared, nervous, funny, and dangerous.” The presentation of composite images were randomized and balanced across participants. All survey data can be found in the S1 Data for this article. This survey protocol was approved by the Lincoln Park Zoo research advisory committee.

### Data analysis

Survey data were analyzed in SPSS (v. 18) using a logistic regression model with forward selection and pair-wise comparisons were made using a chi-square analysis (per [8]). For both the pet and conservation questions, only responses of “yes” or “no” were included in the analysis (per [8,12,13]). There were no differences in responses by species (capuchin, squirrel monkey, ring-tailed lemur), so data were pooled and “species” was dropped from all statistical models. The final models included human (present vs. absent), context (office vs. natural) and human by context interaction.

## Results and Discussion

In response to the first survey question “*Would you consider getting this animal as a pet*?,” 18.7% of respondents stated that they would consider obtaining the pictured primate as a pet (79.5% said they would not consider it and 1.8% were unsure). The breakdown of affirmative responses by condition is presented in Table 1. There was a significant effect of human presence (X^2^ = 10.945, df = 1, p = 0.001) and a human presence by context interaction (X^2^ = 6.44, df = 1, p = 0.011) on likelihood of desiring the primate as a pet. Post-hoc tests revealed significant differences between human-office photos and human-natural photos (X^2^ = 7.68, df = 1, p = 0.006); no human-natural photos (X^2^ = 8.06, df = 1, p = 0.005); and no human-office photos (X^2^ = 7.70, df = 1, p = 0.005).

**Table 1:** The proportion of participants who responded yes to the question, “Would you consider getting this animal as a pet?” when presented with photographs showing a primate pictured with or without a human in a natural or office setting.

	Human	No Human	Total
Natural	0.17	0.16	**0.16**
Office	0.26	0.17	**0.21**
**Total**	**0.21**	**0.16**	

Thus, while the majority of respondents did not want to own the primate, nearly 1 in 5 people answered that they would consider having the displayed primate species as a pet. Consideration of pet ownership was significantly influenced by the image they viewed, with those that viewed the primate in an office setting (i.e. anthropomorphic environment) while in contact with a human, being significantly more likely to desire it as a pet (see Table 1). Prior research on this issue with chimpanzee performers also showed that the presence of a human in the image was associated with increased appeal as a pet [8]. Private ownership of non-human primates is opposed by scientists, zoo professionals, conservationists, and researchers working in the field of primatology citing animal welfare, human health, and public attitude concerns similar to those raised by opponents to their use as performers in the media [14, 15]. Thus, any practice that may increase the public’s perceptions of the acceptability of these animals as pets is important to identify and mitigate in some way.

In response to the second survey question “*Is this species endangered in the wild*?,*”* 69.9% of respondents stated that they were in fact endangered (11.5% said they were not endangered and 18.6% were unsure) (see Table 2). This percentage is similar to that reported when people viewed images of chimpanzees (66%) in the 2008 Ross study [7]. Yet, analyses showed no significant effect of human presence or context on responses to this question as seen with chimpanzee performers [8], nor was there an interaction effect (see Table 2). We speculate that respondents in the present study were potentially primed to believe all primates to be endangered regardless of type or context compared to prior studies due to the rise in public dialog of conservation issues including the use of social media to share information on these topics. This difference in findings between chimpanzee and other primate performers warrants continued inquiry.

**Table 2:** The proportion of participants who responded yes to the question, “Is this species endangered in the wild?” when presented with photographs showing a primate pictured with or without a human in a natural or office setting.

	Human	No Human	Total
Natural	0.88	0.85	**0.86**
Office	0.81	0.90	**0.85**
**Total**	**0.84**	**0.87**	

Interestingly, in looking at responses to the first two survey questions, a proportions test revealed that people who reported wanting the primate as a pet were significantly more likely to believe it was not endangered than those who did not want the primate as a pet (Z = 3.23, p = 0.001). More specifically, those stating they would consider getting the primate as a pet in the first survey question were nearly twice as likely to feel the primate depicted was not endangered. This finding highlights another way in which public attitudes of these species can be influenced by their appearance in the media. Findings from these questions show that depicting non-human primates in an anthropomorphic setting such as an office and in contact with a person can make them more desirable as a pet, which may have the potential to propagate the belief that their wild populations are stable.

**Fig 1:**
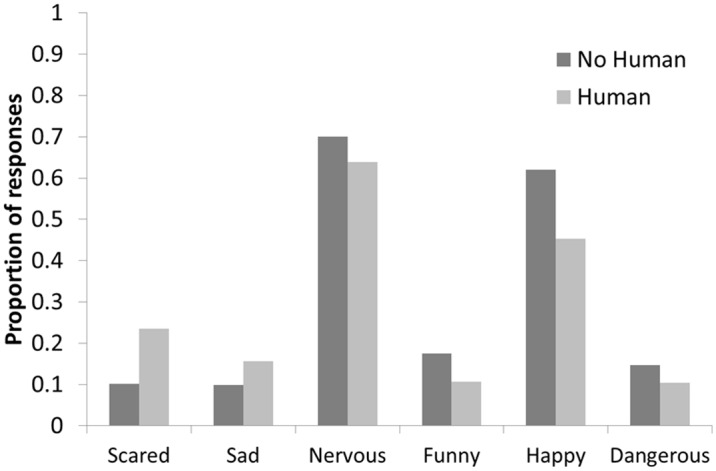
The proportion of respondents who described the primate using the trait when presented with a photo of the primate with or without a human present, averaged across the natural and office settings.

In the final question of the survey, people were asked to circle words that they thought best described the animal in the image and were given the options of “happy, sad, scared, funny, nervous, dangerous.” The most frequent response given regardless of setting and species (> 65%) was that people felt the primate appeared “nervous,” which is important for the advertising and entertainment industry to consider since they are often utilizing these animals as performers for added humor and product likeability (see Fig. 1). Further analysis demonstrated that the presence of a human in the photo significantly increased the proportion of respondents who indicated the primates looked scared (X^2^ = 34.61, df = 1, p < 0.001) and sad (X^2^ = 8.11, df = 1, p = 0.004) and decreased the proportion who indicated the primates looked nervous (X^2^ = 4.83, df = 1, p = 0.028), funny (X^2^ = 10.57, df = 1, p = 0.001), happy (X^2^ = 32.02, df = 1, p < 0.001), and dangerous (X^2^ = 4.76, df = 1, p = 0.029) (see Fig. 1). Therefore, utilizing a primate in conjunction with human actors (regardless of context) appears to have negative impacts on the perceptions and experience of the viewing audience. Further, more people felt the primate was “happy” when shown in the absence of people, and particularly so when depicted in a naturalistic forest habitat, which may reflect public desire for these animals to remain in a natural habitat away from direct human contact. Finally, people were most likely to feel the primate to be “dangerous” when shown in a natural setting with no human present. Thus in that context, people correctly perceive it to be a wild animal, but when placed in contact with people or in an anthropomorphic environment, the animal seemingly becomes less threatening. This has important public safety implications in that people may falsely believe primates they encounter as pets or performers are safe to handle and do not pose the same risks as one encountered in the wild.

## Conclusions

The use of non-human primates as performers is clearly a practice that is in the midst of changing policies and public opinion. Previous studies support the banning of this practice in great apes [1,7,8] and our findings highlight the need for further consideration of the impact of this practice with other non-human primates. Decision-makers in the entertainment and advertising industries should at a minimum consider how the depiction of primates can influence audience mood and perceptions of the conservation status of primates and their desirability as pets. While this work is only a first examination of these issues, the utilization of non-human primates for comedic purposes or to promote a product may have broader consequences than industry representatives have realized to date.

## Supporting Information

S1 Data This file contains the data collected from all survey participants. (XLSX)

## References

[1] Schroepfer KK, Rosati AG, Chartrand T, Hare B (2011) Use of “entertainment” chimpanzees in commercials distorts public perception regarding their conservation status. PLoS ONE. 6(10). (10.1371/journal.pone.0026048). 22022503 PMC3192158

[2] Tomkovick C, Yelkur R, Christians L (2001) The USA’s biggest marketing event keeps getting bigger: an in-depth look at Super Bowl advertising in the 1990s. J. Marketing Comm. 7: 89–108.

[3] Lancendorfer KM, Atkin JL, Reece BB (2008) Animals in advertising: Love dogs? Love the ad. J. Bus. Res. 61: 384–391. (10.1016/j.jbusres.2006.08.011).

[4] International Primatological Society website. Available: http://www.internationalprimatologicalsociety.org/OppositionToTheUseOfNonhumanPrimatesInTheMedia.cfm. Accessed 2015 Jan 23.

[5] Freeman HD, Ross SR (2014) The impact of atypical early histories on pet or performer chimpanzees. PeerJ 2e:579 (10.7717/peerj.579). 10.7717/peerj.579 25279262 PMC4179557

[6] Association of Zoos and Aquariums website. Available: http://www.aza.org/white-paper-apes-in-media-and-commercial-performances/. Accessed 2015 Jan 23.

[7] Ross SR, Lukas KE, Lonsdorf EV, Stoinski TS, Hare B, Shumaker R, et al (2008) Inappropriate use and portrayal of chimpanzees. Science 319: 1487 (10.1126/science.1154490). 18339923

[8] Ross SR, Vreeman VM, Lonsdorf EV (2011) Specific image characteristics influence attitudes about chimpanzee conservation and use as pets. PLoS ONE. 6(7). (10.1371/journal.pone.0022050). 21779372 PMC3135618

[9] The Walt Disney Company website. Available: http://thewaltdisneycompany.com/citizenship/policies/disney%E2%80%99s-use-live-animals-entertainment-policy. Accessed 2015 Jan 23.

[10] Project ChimCARE website. Available: http://www.chimpcare.org/in_the_media/categories/8/. Accessed 2015 Jan 23.

[11] Internet Movie Database website. Available: http://www.imdb.com/name/nm2640714/. Accessed 2015 Jan 23.

[12] Beatty P, Herrmann, D (1995) A framework for evaluating “don’t know” responses in surveys. Proceedings of the Section for Survey Research Methods. Alexandria, VA: American Statistical Association. (American Statistical Association website. Available: http://www.amstat.org/sections/SRMS/Proceedings/papers/1995_175.pdf. Accessed 2015 Jan 23).

[13] Rubin DB, Stern HS, Vehovar V (1995) Handling “don’t know” survey responses: the case of the Slovenian plebiscite. J Am Stat Assoc, 90: 822–828. (10.1080/01621459.1995.10476580)

[14] International Primatological Society website. Available: http://www.internationalprimatologicalsociety.org/PrivateOwnershipOfNonHumanPrimates.cfm. Accessed 2015 Jan 23).

[15] Soulsbury DC, Iossa G, Kennell S, Harris S (2009) The welfare and suitability of primates kept as pets. J. Ap. An. Wel. Sci. 12: 1–20. (10.1080/10888700802536483). 19107661

